# Electronic Bone Growth Stimulators for Augmentation of Osteogenesis in *In Vitro* and *In Vivo* Models: A Narrative Review of Electrical Stimulation Mechanisms and Device Specifications

**DOI:** 10.3389/fbioe.2022.793945

**Published:** 2022-02-14

**Authors:** Peter J. Nicksic, D’Andrea T. Donnelly, Madison Hesse, Simran Bedi, Nishant Verma, Allison J. Seitz, Andrew J. Shoffstall, Kip A. Ludwig, Aaron M. Dingle, Samuel O. Poore

**Affiliations:** ^1^ Division of Plastic Surgery, University of Wisconsin School of Medicine and Public Health, Madison, WI, United States; ^2^ Des Moines University School of Medicine and Health Sciences, Des Moines, IA, United States; ^3^ Department of Biomedical Engineering, University of Wisconsin—Madison, Madison, WI, United States; ^4^ Wisconsin Institute for Translational Neuroengineering (WITNe), Madison, WI, United States; ^5^ Department of Biomedical Engineering, Case Western Reserve University, Cleveland, OH, United States

**Keywords:** electrical stimulation, bone healing, *in vitro*, animal, direct current, pulsed electromagnet field, capacitive coupling

## Abstract

Since the piezoelectric quality of bone was discovered in 1957, scientists have applied exogenous electrical stimulation for the purpose of healing. Despite the efforts made over the past 60 years, electronic bone growth stimulators are not in common clinical use. Reasons for this include high cost and lack of faith in the efficacy of bone growth stimulators on behalf of clinicians. The purpose of this narrative review is to examine the preclinical body of literature supporting electrical stimulation and its effect on bone properties and elucidate gaps in clinical translation with an emphasis on device specifications and mechanisms of action. When examining these studies, trends become apparent. *In vitro* and small animal studies are successful in inducing osteogenesis with all electrical stimulation modalities: direct current, pulsed electromagnetic field, and capacitive coupling. However, large animal studies are largely unsuccessful with the non-invasive modalities. This may be due to issues of scale and thickness of tissue planes with varying levels of resistivity, not present in small animal models. Additionally, it is difficult to draw conclusions from studies due to the varying units of stimulation strength and stimulation protocols and incomplete device specification reporting. To better understand the disconnect between the large and small animal model, the authors recommend increasing scientific rigor for these studies and reporting a novel minimum set of parameters depending on the stimulation modality.

## Introduction

Since Fukada and Yasuda first described the piezoelectric property of bone and its relationship to bone formation (Fukada and Yasuda, 1957)—that bone generates endogenous electrical fields generated by ionically-driven currents in response to mechanical stress—there has been interest in applying exogenous electrical stimulation to stimulate bone healing. There are three modalities of electrical stimulation (ES): direct current electrical stimulation (DCES), capacitive coupling (CC), and inductive coupling. Direct current electrical stimulation is an invasive method of ES in which a cathode is placed directly in contact with the osseous injury. The anode is typically placed in nearby soft tissue, and the current runs between these two electrodes. Capacitive coupling is a noninvasive method of ES in which two electrodes are placed on the opposite sides of the bone, generating an electric field between them. Inductive coupling exists predominantly as pulsed electromagnetic field (PEMF) therapy, a non-invasive methodology in which two solenoids are placed on opposite sides of the bone, parallel to the skin surface. Current is pulsed through the solenoids and generates a magnetic field between them. The magnetic field then, in turn, induces a perpendicular electric field in tissue.

As of 2018, there are 9 FDA-approved electrical bone growth stimulators (EBGS) that are commercially available for the treatment of spinal fusion and fracture nonunion (Khalifeh et al., 2018). There have also been clinical and animal studies performed for a variety of indications including fresh fractures, osteotomies, and treatment of osteoporosis. Despite this, a survey study of orthopedic surgeons in 2020 reported that 68% have never used an EGBS (Bhavsar et al., 2020). When asked what problems are seen with currently available devices, the most common answers were high costs (35%) and inconsistent results (28%) (Bhavsar et al., 2020). To better understand how EBGSs may be improved, a deeper insight of the modality-specific mechanisms, device specifications, and stimulation protocols is imperative.

There have been three recent reviews published on the clinical application of EGBSs (Mollon et al., 2008; Aleem et al., 2016; Khalifeh et al., 2018). These reviews cover ES in the treatment of spinal fusion, nonunion, osteotomies, and acute (or fresh) fractures. Synonymous with fresh fracture, acute fracture refers to a fracture that goes down the normal healing pathway after prompt reduction and fixation. This is opposed to delayed union or nonunion—when the fracture does not heal in the appropriate time after fixation. One meta-analysis of randomized controlled trials for PEMF studies concluded that there was no effect of ES on longbone osseous injuries, but there was substantial heterogeneity between studies likely due to variation in ES treatment protocols (Mollon et al., 2008). A more recent meta-analysis of sham-controlled trials found that ES was effective in reducing nonunion rate at 1 year for all indications—spinal fusion, osteotomies, fresh fractures, and nonunion—(RR = 0.65, 95% CI = 0.53–0.81, *p* = .0001), but there was no significant effect found for any subgroups analyzed except spinal fusion (Aleem et al., 2016). Khalifeh et al. performed a systematic review of all ES studies for the purpose of bone healing and, due to the heterogeneity of the data, was unable to perform a meta-analysis. The study instead categorized the level of evidence in support of treating a specific osseous injury (nonunion, fresh fracture, osteotomy, spinal fusion, and osteonecrosis of the femoral head) by the modality of ES (Khalifeh et al., 2018). The lowest quality evidence was in support of the treatment of nonunion for all modalities, and the highest quality evidence was for CC and PEMF to augment spinal fusion (Khalifeh et al., 2018). The mixed results of these studies highlight the need for standardization of ES treatment protocols and device specifications.

There have been three recent reviews focusing on the mechanisms and applications of ES on bone in preclinical studies (Khatua et al., 2020; Leppik et al., 2020; Vicenti et al., 2020). Khatua et al. emphasizes the piezoelectric and magnetoelectric biocomposites and their mechanisms of osteoproliferation *in vitro* (Khatua et al., 2020). Leppik et al. reviews the proposed mechanisms of ES-induced osteoproliferation *in vitro* including cellular attachment, differentiation, alignment, and migration on osteoconductive scaffolds (Leppik et al., 2020). Vicenti et al. categorizes the specifications used in PEMF studies in preclinical and clinical case series and trials (Vicenti et al., 2020).

The purpose of this review is twofold: first, we aim to catalog key findings in *in vitro*, small animal, and large animal studies, and second, we discuss these findings with an emphasis on device specifications and stimulation protocols, identifying gaps in reporting that need to be addressed in future studies to better understand mechanism and efficacy. To do this, we queried the MEDLINE database for relevant search terms, including combinations of “[(electrical stimulation) AND (bone) AND (in vitro)] OR [(electrical stimulation) AND (bone) AND (*in vivo*)].” We selected records pertaining to the use of electrical stimulation to augment osteogenesis, including *in vitro* studies, fracture healing, delayed and nonunion treatment, osteotomies, osteoporosis, bone grafting, implant integration, and spinal fusion. All human studies and those not available in English language were excluded. Our review includes 50 studies—19 *in vitro* studies, 18 small animal studies, and 13 large animal studies. Of the *in vitro* studies, 8 are for DCES, 8 are for PEMF, and 3 are for CC (Table 1). There are 8 small animal studies for DCES, 6 for PEMF, and 4 for CC (Table 2). There are 8 DCES studies in large animals, 3 PEMF studies, and 2 CC studies (Table 3).

**TABLE 1:** This table presents the cell line studied, stimulation protocols, device specifications, and outcomes of *in vitro* studies included in our review.

Author	Year	Cell line	Protocol	ES Specs	Outcome
DCES					
Okihana et al.	1988	Rabbit chondrocyte	Continuous for 7 days	Platinum cathode, 0.1, 1 or 10 µA	Proteoglycan synthesis and nucleotide incorporation highest at 1 µA (*p* < .025, *p* < .01)
Dauben et al.	2016	Human osteoblasts	45 min, 3 times per day for 3 days	Titanium cathode, 0.2 and 1.4 V_RMS_, 20 Hz*	1.4 Vrms inceased OC transcription (*p* = .0148), 0.2 Vrms increased procollagen type 1 (*p* = .0051)
Leppik et al.	2018	Rat mesenchymal stem cells	1 h/day for 3 weeks	Stainless steel cathode, 100 V/m*	Increase ALP expression at days 7 and 14 (*p* < .05), TBF-β increased at day 7 (*p* < .05), Osteopontin and Calmodulin increased at all time points (*p* < .05)
Jing et al.	2019	Rat bone mesenchymal stromal cells	3 h/day for 21 days	Lactide polymer cathode, 75 V/m*	Increased ALP, COL-1 mRNA and calcium deposition (*p* < .05)
Li et al.	2019	Mouse fibroblasts	Once for 2 h	Silver cathode, 100 mV/mm*	Increased intracellular calcium (*p* < .01) and increased expression of proliferation and cell cycle related proteins 24 h later after ES treatment (*p* < .001)
Portan et al.	2019	Human osteoblasts	20 min to 2 h/day for 3 days	Titanium cathode, 0.3 V or 1 V, 1 Hz to 10 MHz*	Group of 0.3 V, 2 h/day for 3 days increased ALP/total protein ratio (*p* < .05)
Srirussamee et al.	2019	Mouse macrophages and preosteoblasts	1–2 h/day for 3 days	Platinum cathode, 2.2 V, 1 V/cm, 0.07 ± 0.01 mA	Group stimulated 2 h/day induced BMP2 and SPP1 mRNA expressions (*p* < .05), ES reduced overall metabolic activity of both cell types
PEMF					
Shankar et al.	1998	Rat osteoclasts	Once for 18 h	15 Hz, 4.5 ms assymetric pulses, 18G	1.8 mT: 2 fold increase in bone resorption (*p* < .009)
Lohmann et al.	2000	Human osteoblast-like cells	8 h/day for 1, 2, or 4 days	5 ms bursts of 20 pulses, 15 Hz, 18G	Stimulated cells had greater collagen synthesis and osteocalcin production, prostaglandin E2 was reduced, growth factor β1 increased, higher levels of alkaline phosphatase
Tsai et al.	2009	Human mesenchymal stem cells	2 h/day for 10 days	0.13 mT, 7.5 Hz, Efield: 2 mV/cm, 300 µs quasi-rectangular pulses	Day 7: control had 84% more cells (*p* < .05), ALP increased in PEMF by 82% (*p* < .01) Day 10: PEMF group had 62% more cells (*p* < .05), 123% increase in cells in control
Lin et al.	2011	Mice osteoblasts	Once for 9 h	1.5 mT, 2.5 mV, 75 Hz, pulse duration of 1.3 ms	Day 7: stimulated cells had 36% more chance of being viable, 23% more total DNA, 40% less ALP activity, increase COL1, no change in osteocalcin (*p* < .05)
Zhong et al.	2012	Human Bone marrow stromal cells	8 h/day for 12 days	0.5 mT, 50 Hz	Day 10: stimulation increased cell proliferation (*p* = .0312) increased ALP (*p* = .01), increased collage 1 gene mRNA (*p* = .0001)
Barnaba et al.	2013	Human osteoblasts	Continuous for 3, 7 or 10 days	0.4 mT 14.9 Hz	Cell stimulated for 3,7, and 10 days increased cell proliferation by 1.8% 29 and 55.5%, day 10 also had 3 times as much ALP activity in stimulated cells
Grunert et al.	2014	Human osteoblasts	45 min/day, 3 times per day for 3 days	electric potentials around screw: 0.514–0.796 V, electric potentials on top and bottom surfaces of the scaffold: 0.38–0.43 V, 20 Hz, magnetic flux density: 3 mT	Stimulation increased COL1 synthesis 3 fold (*p* < .05) and was metabolized less (*p* = .026)
Escobar et al.	2020	Rat chondrocytes	1,3 or 5 h, 4 times per day for 8 days	2 mT, 100 V AC (input), 60 Hz, 6 V (output)	No difference in cell proliferation
CC					
Brighton et al.	1976	Rat epiphyseal plate cells	Continuous for 10 days	0, 500, 1,000, 1,500, 2,000, 3,000 V/cm	500 V/cm and higher stimulated epiphyseal elongation, 1500 V/cm greatest elongation (*p* < .0005)
Brighton et al.	1984	Bovine chondrocytes	Continuous for 24 h	0, 10, 100, 250, 1000 V 60 Hz, 0.37 μA/mm	Increased nucleotide incorporation for 250 V group in 1% NCBS (*p* < .01) and to a lesser extent at 10% NCBS, decreased nucleotide incorporation at 10 V peak-to-peak (*p* < .01)
Stephan et al.	2020	Human mesenchymal osteoblast-like cells	Continuous for 3 days	2.5–3.5 mV/m or 0.24–0.35 mV/m, 60 kHz	0.1 Vrms increased metabolic rate (*p* = .002), reduced procollagen type 1 propeptide (*p* = .048), increased TIMP1 (*p* = .017), increased OPG mRNA (*p* = .005)

Asterisks (*) denote incomplete device specifications. Green fill denotes significant results. Red fill denotes non-significant results.

**TABLE 2:** This table presents the animal model, weight and age, animal sex, osseous injury, fixation stimulation protocol, device specifications, and outcomes of the small animal studies included in our review.

Author	Year	Animal	Weight and age	Sex	Osseous injury	Fixation	Protocol	ES specifications	Outcome
DCES									
Petersson et al.	1982	Rabbit	NR, adult	NR	Intramedullary femur	NA	Continuous for 28 days	Stainless-steel cathode, 20 µA	No difference in bone mineral density or histologic measurement of cortical thickness
Spadaro et al.	1982	Rabbit	2 kg, adult	Male	Intramedullary femur	NA	Continuous for 21 days	Platinum, stainless-steel, titanium, chromium-cobalt, gold, and silver cathodes, 0.02 and 0.2 μA/mm^2^	Different cathode materials stimulate osteogensis optimally at different current densities
Zimmerman et al.	1984	Rabbit	3.5–5 kg, adult	Male	Intramedullary tibia	NA	Continuous for 21 days	Carbon fiber cathode, 1, 5, and 20 μA	Greatest new bone in 1 μA group, most fibrous tissue in 20 μA group
Rubinacci et al.	1988	Rabibit	3.4 ± 0.2 kg, adult	Male	Intramedullary tibia	NA	Continous for 14 days	Stainless-steel cathode, 20 μA	Increase in periosteal bone formation (*p* < .0001) measured by histomorphology
Shafer et al.	1995	Rabbit	5.3 ± 0.5 kg, adult	NR	Mandibular dental implant integration (3.75 × 7.00 mm)	NA	Continuous for 28 days	Titanium dental implant, 7.5 μA	No difference in torque-to-failure or histomorphologic analysis
France et al.	2001	Rabbit	4.3 kg, adult	NR	Lumbar fusion (4 cm^3^)	NR	Continuous for 35 days	Titanium cathodes, 20 and 60 μA	Increased radiograph fusion grades (*p* < .04), higher thresholds of biomechanical failure (*p* < .02) in 60 μA group, no difference in 20 μA group
Nakajima et al.	2010	Rat	NR, 12-week-old	Male	Tibial osteotomy	21G needle IM fixation	20 min/day for 3 weeks	Stainless-steel cathode, 50Hz, 20 μA	Increased bone formation by histology (*p* < .05), callus formation measured radiograph (*p* < .05), increased maximal load by biomechanical testing (*p* < .05)
Leppik et al.	2018	Rat	NR, 9-week-old	Male	Femur gap osteotomy (5 mm)	microplate	1 h/day for 8 weeks	Stainless steel cathode, 0.1–0.2 μA	Increased neovascularity and endochondral bone formation (*p* < .05)
PEMF									
Guizzardi et al.	1994	Rat	350–400 g, adult	Male	Lumbar fusion (no defect size reported)	NR	12 h/day for 8 weeks	2.5 mV*	Histomorphological analysis: no significant difference. Qualitative osteoblastic prolifferation increased at week 8
Buzza et al.	2003	Rabbit	NR, adult	Female	Tibial dental implant integration (2.6 × 6.0 mm)	NA	30 min/day, 5 days/wk for 42 days	85 μsec pulse width, 25 Hz freq*	No difference in torque-to-failure
Van der Jagt et al.	2012	Rat	220 g, 20-weeks-old	Female	Osteoporosis (ovariectomy)	NA	2–4 h/day, 5 days/wk for 6 weeks	0.1 mT 15 Hz, 0.1 mT 15 Hz w/5 min on/off cycles, 0.1 mT 15 Hz w/added 150 Hz noise, 0.1 mT 7.5 Hz	No difference in bone densitity found using microCT scanning
Atalay et al.	2015	Rat	NR, 12-weeks-old	Male	Acute femur fracture (transverse, no gap)	4-hole microplate	6 h/day, 7 days/wk for 30 days	1.5 ± 0.2 mT, 50 Hz	Improved volumes of osteoblastic material by histomorphologic analysis at 21 and 30 days (*p* < .05)
Liu et al.	2020	Rat	NR, 3-months-old	Male	Femur bone wound (unicortical, 1 mm wide)	NA	2 h/day for 7 days	1 mT, 5 mT, and 10 mT, 15 Hz	Biomechanical measurements showed fracture load higher in 5 and 10 mT (*p* < .05), 1 mT not significant
Androjna et al.	2021	Rat	NR, 6-months-old	Female	Osteoporosis (ovariectomy)	NA	3 h/day, 7 days/wk from weeks 4–10 post-operatively	0.41 mT, 1.2 mT, 4.1 mT, and 12.1 mT*	No difference in bone mineral density found using microCT scanning
CC									
Marino et al.	1979	Rat	NR, 21-days-old	Male	Fibular osteotomy (transverse, no gap)	None	Continuous for 14 days	5000 V/m, 60 Hz	Lower histomorphologic grades of healing 21 days s/p (*p* < .01)
Brighton et al.	1988	Rat	NR, 3-weeks-old	Male	Osteoporosis (sciatic neurectomy)	NA	Continuous for 12 days	5 V, 60 Hz*	Increased bone mineral density by wet weight (*p* < .01). No difference in rate of bone resorption and did not prevent osteoporosis
Medalha et al.	2010	Rat	NR, 8-weeks-old	Female	Osteoporosis (T9 spinal cord transection)	NA	20 min, 3 times/week for 4 weeks.	1.5 Mhz*	No difference in maximal load or densitometry, improved cortical thickness by morphometric analysis (*p* < .05)
Manjhi et al.	2010	Rat	230–250 g, adult	Female	Osteoporosis (ovariectomy)	NA	2 h/day for 60 days	10 V, 16 Hz*	Bone mineral content and density increased by X-ray diffraction (*p* < .01, *p* < .001)

Asterisks (*) denote incomplete device specifications. Green fill denotes significant results. Red fill denotes non-significant results. Yellow fill denotes mixed results. NR = not recorded. NA = not applicable.

**TABLE 3:** This table presents the animal model, weight and age, sex, osseous injury, fixation, stimulation protocol, device specifications, and outcomes of the large animal studies included in our review.

Author	Year	Animal	Weight and age	Sex	Osseous injury	Fixation	Protocol	ES specifications	Outcome
DCES									
Paterson et al.	1977	Dog	NR, NR	NR	Tibial gap osteotomy (1.5 cm)	Intramedullary rod	Continuous for 28 days	Stainless-steel cathode, 20 µA	Improved healing (*p* = .05) measured by histological analysis
Paterson et al.	1977	Dog	NR, NR	NR	Tibial gap osteotomy (1.5 cm)	Intramedullary rod	Continuous for 56 days	Stainless-steel cathode, 20 µA	Improved fibrous tissue and endochondral ossification measured by histology (*p* = .042) and visual assessment score of bone healing (*p* < .01)
Srivastava et al.	1982	Dog	NR, NR	NR	Tibial fracture	Long leg cast	Continuous*	Stainless steel cathode with long-leg plaster cast, 20 µA	Improved healing measured by histomorphologic analysis*
Chakkalakal et al.	1990	Dog	18–22 kg, adult	NR	Radial osteotomy (transverse, gapless)	4-hole teflon plate	Continuous*	Teflon-insulated copper cathodes, 0.1–100 µA	improved healing at 1, 7, and 13 µA measured by improved rigidity via biomechanical testing (*p* < .001, *p* < .002, *p* < .009), and histomorphologic analysis (*p* < .021, *p* < .004, *p* < .025, respectively)
Nerubay et al.	1986	Porcine	NR, 1-month-old	NR	Lumbar fusion (L5-L6 disc space)	None	Continuous for 0–56 days	Stainless-steel cathodes, 20 µA	Improved healing measured with radiographic fusion score (*p* = .037), osteoblastic activity by histomorphologic scoring (*p* < .01)
Toth et al.	2000	Sheep	NR, adult	NR	Posterior lumbar spinal fusion (L4-5 disc space)	Fixation cage	Continuous for 4 months	No cathode material reported, 40 and 100 µA groups	100 µA increased fusion score (*p* = .003). Biomechanical testing increased flexion to failure (*p* < .029)
Cook et al.	2004	Rhesus macaques	11.3 ± 3.6 kg, adult	1:1 Male to Female	Anterior spinal fusion (L5-6 disc space)	None	Continuous for 84 days	Titanium cathode; low current density (5.4 μA/cm^2^), high current density (19.6 μA/cm^2^)	Reduced fusion time by radiographic fusion grade (*p* = .0001). Higher fusion rate measured by computed tomography (*p* = .0314)
Lindsey et al.	1987	Dog	23–32 kg, adult	NR	Autologous bone graft in femur (12 × 13 mm)	6-hole stainless steel plate	Continuous for 56 days	Coiled titanium cathode, 20 µA constant for 8 weeks	No difference in torque-to-failure
PEMF									
Miller et al.	1984	Dog	17–25 kg, 2-5-years-old	Male	Fibula bone graft (4 cm cortical segment)	None	20 h/day*	15 Hz, 5 ms bursts in an asymmetrical waveform*	no improvement measured by biomechanical testing and histomorphologic assessment
Inoue et al.	2002	Dog	NR, adult	Male	Tibial osteotomy (transverse, 2 mm gap)	External fixator	1 h/day, 7 days/wk for 4 weeks	0.2 mT, 1.5 Hz*	Improved healing measured by histomorphologic scoring (*p* < .04, *p* < .05)
Law et al.	1985	Sheep	45 kg, adult	NR	Tibial osteotomy (transverse, no gap)	6-hole nylon plate	24 h/day, 7 day/wk for 6 weeks	1.1 mT*	No improvement measured by histology staining and radiographs
CC									
Pepper et al.	1996	Dog	11.7 ± 2.3 kg, adult	Male	Tibial osteotomy (transverse, no gap)	Distraction osteogenesis	Continuous for 28 days	60 kHz, 3–6.3 V, 5–10 μA*	No improvement measured by biomechanical, histologic, and radiographic analyses
Muttini et al.	2014	Sheep	62–70 kg, 2-years-old	NR	Tibial osteotomy (transverse, no gap)	External fixator	12 h/a day	1500 μA, 12.5 Hz*	Increased callus maturation measured by histology (*p* < .0001). Active stimulation group had increased opacity measured by quantitative radiodensity analysis (*p* < .0043)

Asterisks (*) denote incomplete device specifications. Green fill denotes significant results. Red fill denotes non-significant results. Yellow fill denotes mixed results. NR = not recorded.

## *In Vitro* Studies

*In vitro* experiments have been used to describe the effects of ES on many different cell types, including osteoblasts and chondrocytes (Table 1). Many studies were performed on osteoblasts and their precursors, offering information about cell migration and proliferation in the bone healing process (Dauben et al., 2016; Leppik et al., 2018; Jing et al., 2019; Portan et al., 2019; Srirussamee et al., 2019). Chondrocytes from the growth physis or callus are studied to determine their differentiation to osteoblasts and extracellular matrix deposition (Brighton et al., 1976; Okihana and Shimomura, 1988). Articular chondrocytes have also been studied to determine if ES can induce cartilage regeneration (Brighton et al., 1984). The primary goals of these studies are to describe the cellular mechanisms of ES on osteoproliferation and to define ES specifications and protocols at which osteoproliferation is most active. Being able to describe the effects of ES at a cellular level can help elucidate clinical applications. One roadblock to synthesis of information for translation into *in vivo* models and eventual clinical application is significant variation in outcome measures and device specifications.

### Differences in Outcome Measures, Device Specifications, and Units of ES Make Interpreting and Reproducing *In Vitro* Studies Difficult

Many *in vitro* studies use different cellular markers of osteoproliferation, making it difficult to compare results between studies. One example of a cellular marker for osteogenesis used in these studies is glycosaminoglycan (GAG) synthesis, as it is known to form bone at the epiphyseal plate (Brighton et al., 1984). This has been measured by [^35^H]thymidine and [^3^S]sulfate uptake in chondrocytes and safranin-O staining (Brighton et al., 1984). Additionally, procollagen protein synthesis is a marker of collagen 1 (COL-1), which is a key component to bone formation, and alkaline phosphatase (ALP) is a biomarker for osteoblasts (Stephan et al., 2020). Procollagen protein synthesis has been measured using the biomarker type I C-terminal collagen propeptide (CICP) via ELISA (Stephan et al., 2020). The ALP gene has been quantified using p-nitrophenyl phosphate (pNpp) with microplate analysis at a wavelength of 405 nm (Stephan et al., 2020). Another marker for osteogenesis is intracellular calcium levels, which contribute to cytoskeletal organization, cell motility, and cell growth and has been measured using flow cytometry (Li et al., 2019). Upregulation of bone morphogenic protein 2 (BMP2) is often used to describe osteoproliferation on a cellular level as it is responsible for differentiation of osteoprogenitor cells and has been quantified with a reverse transcriptase quantitative polymerase chain reaction (Srirussamee et al., 2019). Cell metabolic activity for osteoblast precursors has been measured with a resazurin assay (Srirussamee et al., 2019).

In addition to the many types of cellular markers used for osteogenesis, there are other trends in *in vitro* studies that became apparent during our review: firstly, *in vitro* studies typically demonstrate a dose-dependent relationship with stimulation and some marker of osteogenesis. However, the rationale with which the levels of stimulation are selected is often not explained. Next, the custom device specifications are often incomplete and not reproducible based on the information provided. Finally, the units of measurement for electrical stimulation are not convertible between many studies.

### Capacitive Coupling Studies—*In Vitro* Models

Capacitive coupling was the first ES modality studied *in vitro* when Brighton et al. subjected cells within a rat epiphyseal plate model to varying strengths of electrical field—0, 500, 1,000, 1,500, 2,000, 2,500, and 3000 V/cm—constantly for 10 days and measured total epiphyseal, osseous, and cartilaginous cellular elongation (Brighton et al., 1976). All electrical field strengths greater than 500 V/cm stimulated total epiphyseal, osseous, and cartilaginous elongation more than unstimulated controls, but the 1,500 V/cm demonstrated the greatest amount of elongation in all variables measured (*p* < .0005). In 1984, Brighton et al. administered CC constantly for 24 h to isolated bovine articular chondrocytes at varying voltages—0, 10, 100, 250, and 1,000 V—at 60 Hz (Brighton et al., 1984). No electric field strengths were reported. The 250 V group increased nucleotide incorporation when compared to other groups (*p* < .01). More recently, Stephan et al. measured cellular markers of osteoproliferation in isolated human mesenchymal osteoblast-like cells (Stephan et al., 2020). The groups analyzed were cells treated with a sham stimulator, cells treated with 1V_RMS_ (root mean squared), equivalent to 0.25–0.35 V/cm, and cells treated with 0.1V_RMS_, equivalent to 0.025–0.035 V/cm. The authors found that the 1V_RMS_ group had a higher metabolic rate (*p* = .002), reduced CICP (*p* = .048), increased tissue inhibitor of metalloprotease 1 (*p* = .017), and increased osteoprotegrin mRNA (*p* = .005). There was no significant difference between ALP and osteocalcin (OC) mRNA between groups.

### Direct Current Studies—*In Vitro* Models

Direct current electrical stimulation has also been tested in an *in vitro* model. In 1988, Okihana et al. examined the effect of platinum-cathode DCES at 0, 0.1, 1, and 10 μA constantly for 1 week on rabbit growth chondrocyte proteoglycan synthesis and nucleotide incorporation (Okihana and Shimomura, 1988). At 7 days, proteoglycan synthesis and nucleotide incorporation were highest at 1 μA (*p* < .025, *p* < .01, respectively) when compared to unstimulated controls. The 0.1 and 10 μA groups demonstrated no significant effects.

#### DCES at Varying Doses Improves Differentiation of Osteocyte Precursors

More recently, Dauben et al. studied the effects of titanium-cathode DCES at 0.2 and 1.4 V_RMS_ on osteoprolific genes in human osteoblasts (Dauben et al., 2016). No amperage values were given. With stimulation for 45 min, 3 times per day for 3 days, the 1.4 V_RMS_ group increased OC transcription more than the 0.2 V_RMS_ group (*p* = .0418), but procollagen type 1 synthesis increased when treated with 0.2 V_RMS_ when compared with 1.4 V_RMS_ (*p* = .0051). They also demonstrated that metabolic activity, ALP and COL-1 did not significantly change based on voltage. Jing et al*.* conducted a similar study and found that ALP, COL-1 mRNA, and calcium deposition all increased compared to unstimulated controls when rat osteoblasts were stimulated with lactide polymer cathodes at 0.75 V/cm, 3 h per day for 21 days (*p* < .05) (Jing et al., 2019). No amperage values were given. Leppik et al. treated rat adipose-derived mesenchymal stem cells on a β-tricalciumphosphate scaffold with 100 V/m stimulation for 1 h/day for 3 weeks. In the stimulation group, there an increase in Osteopontin and Calmodulin at all timepoints, TGF-β at 7 days, and ALP at days 7 and 14 (*p* < .05 for all) (Leppik et al., 2018). Portan et al. found that human osteoblasts stimulated with titanium cathodes at 0.3 V, 3 h per day for 3 days, significantly increased ALP/total protein ratio when compared to unstimulated controls (*p* < .05) and the osteoblasts assumed a physiologic morphology and elongated while being stimulated (Portan et al., 2019), demonstrating improved differentiation than controls. Given the different units of ES dosing and outcome measures between these studies, it is difficult to determine an optimal ES protocol to stimulate osteogenesis in osteoblasts.

Direct current electrical stimulation studies have also been conducted on macrophage and fibroblast cell lines. Srirussamee et al. demonstrated that mouse macrophages and preosteoblasts stimulated with a platinum cathode constantly at 70 μA for 2 h per day for 3 days induced BMP2 and secreted phosphoprotein 1 mRNA expressions significantly more than unstimulated cells (*p* < .05) and both cell lines were at the highest concentration between the anode and cathode within the well (Srirussamee et al., 2019). Li et al. found that mouse fibroblasts stimulated a single time with silver electrodes at 100 V/cm for 2 h had increased intracellular calcium by flow cytometry when compared to unstimulated cells (*p* < .01) (Li et al., 2019). The variability of measurement units in ES strength in DCES *in vitro* studies—amperes, volts, V_RMS_, and electrical field—and the inadequate information to convert between them, make it difficult to interpret the wide range of proposed cellular effects of DCES.

### Pulsed Electromagnetic Field Studies—*In Vitro* Models

There have also been many PEMF *in vitro* studies performed on a range of cell lines. Shankar et al. stimulated rat osteoclasts with PEMF specifications of 1.8 mT, 4.5 ms asymmetric pulses (no frequency reported) and demonstrated a two-fold increase in bone resorption compared to unstimulated controls as measured by decreased osteoclastic excavations under scanning electron microscopy (*p* < .009) (Shankar et al., 1998). Lohmann et al. found that human osteoblast-like cells stimulated with 5 ms bursts of 20 pulses repeating at 15 Hz with a magnetic field of 1.8 mT increased markers for differentiation and ECM production, protein synthesis (*p* < .05) (Lohmann et al., 2000). [^3^H]thymidine incorporation was decreased in treated groups by 37% on day two (*p* < .05). Alkaline phosphatase reached a maximum activity on day 1, and percentage of collagen decreased more slowly when treated with PEMF (*p* < .05). Tsai et al. stimulated human mesenchymal stem cells at 0.13 mT, 7.5 Hz for 10 days (Tsai et al., 2009). The control group had 84% more cells at day 7 (*p* < .05), but the PEMF group had 62% more cells by day 10 (*p* < .05), suggesting that PEMF stimulates cell proliferation between days 7 and 10. Alkaline phosphatase activity was increased by 82% in PEMF group at day 7 (*p* < .01) and increased by 123% in the control group at day 10 (*p* < .05), with the conclusion that PEMF stimulation results in earlier ALP activity (Tsai et al., 2009).

Lin et al. demonstrated that at day 7 mice osteoblasts stimulated for 9 h at 1.5 mT at 75 Hz were 36% more likely to be viable, and stimulated cells increased total DNA by 23%, 40% decreased ALP activity, and increased COL-1 expression (*p* < .05) (Lin and Lin, 2011). Zhong et al. found that human bone marrow stromal cells stimulated at 0.5 mT, 50 Hz had increased cell proliferation (*p* = .0312), increased ALP (*p* = .01), and increased COL-1 mRNA (*p* = .0001) at day 10 compared to unstimulated controls (Zhong et al., 2012). Barnaba et al. found that, when human osteoblasts were stimulated with 0.4 mT at 4.9 Hz for 3, 7 and 10 days, cell proliferation increased by 1.8, 29 and 55.5% respectively compared to unstimulated cells, and ALP activity increased to nearly three times the control by day 10 (*p* < .05) (Barnaba et al., 2013). In a similar study, Grunert *et al.* demonstrated that when human osteoblasts were stimulated with PEMF at 3 mT, 20 Hz for 45 min, 3 times per day for 3 days there was a three-fold increase in COL-1 synthesis (*p* < .05) and were more metabolically active than unstimulated cells (*p* < .05) (Grunert et al., 2014). Escobar et al. found that, when rat chondrocytes were stimulated at 2 mT (no frequency reported) for 1, 3, and 5 h every 6 h for 8 days, there was no significant difference in cell proliferation between groups (Escobar et al., 2020). Despite the differences in magnetic field strength, frequency, and length of therapy, there appears to be a common thread of earlier ALP upregulation and increased COL-1 synthesis when cell lines are treated with PEMF.

## Small Animal Studies

In the field of ES for the purpose of bone healing, small animal studies consist entirely of rabbit and rat models. In our review, we include 11 rat model studies and seven rabbit model studies. These studies are useful because they offer the ability to have larger groups to achieve statistical significance for a given effect without constraints of managing large animals like sheep or dogs. However, there are issues that arise in the clinically relevant translation of small animals. For example, the scale and the relatively superficial nature of rat and rabbit long bones eliminate problems with delivery of ES to the fracture site. These small animal models still offer helpful insight in both the mechanism and appropriate target specifications of ES to induce osteogenesis (Table 2). Despite the relative success seen in small animal studies, common issues seen in these studies are inconvertible units of measurement used to describe the dose of ES. For example, describing the strength of a PEMF-induced electrical field in volts does not allow the reader to compare magnetic field strength of PEMF devices in other studies. Other studies use incomplete device specifications that make it impossible to reproduce results, like using a custom DCES device but not reporting the cathode material. Finally, studies lack experimental validation that the custom devices are functioning according to theoretical models, like making a CC device but not measuring the electric field between the electrodes at various points.

### Direct Current Electrical Stimulation Studies—Small Animal Models

#### More Negative Registered Surface Potentials Associated With Healing Bone

Since Brighton et al. published the successful treatment of nonunions with DCES in humans, there have been attempts to further explore this effect in small animal models (Brighton et al., 1977). An early study attempted to characterize the change in endogenous bioelectric potentials that occur during normal long bone healing in a rat tibial fracture model and found that, from the day of fracture to 30 days post-injury, the healing callus maintained a more negative registered potential, between 3 and 6 mV more negative than uninjured controls (Stern and Yageya, 1980).

To further clarify the relationship between negative bioelectric potentials, bone healing, and DCES, Rubinacci et al. recorded surface potentials with continuous, 20 μA intramedullary DCES with a stainless steel cathode for 14 days in an tibial rabbit model and correlated these values to quantitative bone formation over time (Rubinacci et al., 1988). While current was applied, a negative spike in bioelectric potential was measured directly over the cathode, which was absent in the control and current-off state. This change in potential was significantly associated with an increase in periosteal bone formation (*p* < .0001), measured by histomorphology (Rubinacci et al., 1988). A similar study was conducted by Petersson *et al.* in which an intramedullary stainless steel cathode was used to administer 20 μA current continuously for 28 days within rabbit femurs (Petersson et al., 1982). However, there was no significant difference between sham and active stimulator groups for radioisotope uptake or histologic analysis of bone formation by total cortical thickness (Petersson et al., 1982).

In Rubinacci et al., the combined thickness of medullary and periosteal bone was not statistically significant between groups, as the control group had some new bone formed around the inactive cathode, presumably due to the inflammatory process of micromotion of the cathode within the medullary canal (Rubinacci et al., 1988). The effect of trauma-induced osteogenesis has been demonstrated to be larger in the well-vascularized femur, which is completely incased in muscle with robust blood supply, when compared to the relatively poorly vascularized tibia, which has no muscle on the anteromedial surface (Spadaro, 1982; Brumback, 1996). This difference in vascularity may explain the lack of significant results in Petersson et al., (Petersson et al., 1982).

#### Cathode Material and Current Density are Important Variables in DCES

In 1982, Spadaro et al. explored the capacity of different electrode cathode materials to induce cancellous osteogenesis in an intramedullary rabbit femur model and found that stainless steel and titanium cathodes performed best at 0.2 μA/mm^2^ current density, while platinum cathodes performed best at 0.02 μA/mm^2^ (Spadaro, 1982). These results agree with earlier studies that aimed to elucidate the mechanism of DCES-induced osteogenesis; That is, the electrochemical reactions near the cathode deplete intracellular oxygen to form free radicals, which stimulate bone formation (Spadaro and Becker, 1979). Spadaro et al. concluded that it is not simply a specific current density, but rather specific concentrations of faradic products and microenvironmental changes in pH and oxygen partial pressures—dependent upon the cathode material and current density—which, at least in part, induce osteogenesis (Spadaro, 1982). More data to support this hypothesis was found in a later study by Zimmerman et al., that used carbon filaments as a cathode for DCES in a rabbit tibial intramedullary model (Zimmerman et al., 1984). The rabbit tibia was stimulated for 21 days continuously at 1, 5, and 20 μA, and it was found that the greatest amount of osteogenesis occurred in the 1 μA group with the largest amount of fibrous tissue generated in the 20 μA group (Zimmerman et al., 1984). That is, the carbon cathode produced the most new bone with a current density of 1.0 × 10^−3^ μA/mm^2^, a fraction of what was found to be adequate to stimulate osteogenesis with other materials (Spadaro, 1982).

#### DCES Improves Osteogenesis, Radiographic Union, and Biomechanical Thresholds for a Variety of Indications

Small animals have been used to investigate the effect of DCES on spinal fusion, bone healing, and implant integration. In a transverse, gapless tibial osteotomy model of 30 12-week-old, male Wistar rats, DCES (50 Hz, 20 μA) administered after intramedullary fixation with 21-gauge needle by a stainless steel cathode for 20 min/day for 3 weeks. The active group demonstrated improved osteogenesis at 3 weeks post-injury (*p* < .05), callus formation measured radiographically at 6 weeks (*p* < .05), and biomechanical testing at 6 weeks (*p* < .05) (Nakajima et al., 2010). France et al. also demonstrated DCES to improve lumbar posterior fusion in a rabbit model (France et al., 2001). The groups analyzed were posterior fusions with a sham stimulator, low current (20 μA stimulator), and high current (60 μA stimulator). No description of cathode dimensions or geometry was offered. The stimulator groups were administered continuous DC with titanium cathodes from post-operative day 0–5 weeks. The high current group demonstrated increased radiographic grades of fusion (*p* < .04) and higher thresholds of biomechanical failure (*p* < .02) at 5 weeks when compared to the sham group, whereas the low current group was not significantly different (France et al., 2001). Shafer et al. examined the effect of continuous 7.5 μA DCES on osteointegration of a titanium dental implant in a rabbit mandible model (Shafer et al., 1995). There was no difference in required removal torque or histological analysis of osteogenesis between the active and sham stimulator groups at 28 days. These studies offer helpful insight into the range of target amperage and appropriate cathode materials required to stimulate osteogenesis.

Leppik et al. applied 0.1–0.2 μA, 100 V/m for 1 h per day through a custom-built stimulator with 10 cm stainless steel cathode (0.228 mm, insulated) to treat a 5 mm femur defect in 81 nine-week-old, male Sprague Dawley rats. The femur defects were fixed with a microplate and filled with a β-tricalciumphosphate scaffold and impregnated with adipose-derived mesenchymal stem cells. At 8 weeks post-surgery, the stimulation group demonstrated more robust callus formation (*p* < .05) and higher scores for neovascularity (*p* < .05) than unstimulated groups (Leppik et al., 2018).

### Pulsed Electromagnetic Field—Small Animal Studies

#### PEMF Yields Mixed Results for Reducing Bone Resorption in Osteoporosis Models

In addition to DCES, multiple small animal studies have been performed to elucidate the efficacy of PEMF for a wide range of indications. Van der Jagt et al. investigated different PEMF protocols’ ability to reduce bone loss in 20 ovariectomized rats. This experimental design included five groups: sham stimulator control group, stimulator group (0.1 mT magnetic field, 5 ms bursts, 5 μs pulse, and 15 Hz for 2 h per day), stimulator group with the same specifications but 5 min on/off cycles for 4 h per day, stimulator group with the same basic specifications and an added random 50–150 Hz 0.1 mT magnetic field for 2 h per day, and a stimulator group (0.1 mT magnetic field, 7.5 Hz, and 0.3 ms pulse) (van der Jagt et al., 2012), All groups were stimulated 5 days per week for 6 weeks. No difference in bone density was seen between groups by microCT at 6 weeks. A similar study was later performed in which the rats were ovariectomized 4 weeks prior to receiving treatment, and osteoporotic status was verified by microCT prior to randomization into the study (Androjna et al., 2021). The PEMF configuration was such that the small cylindrical container in which the rats were housed during ES did not vary more than 10 percent in magnetic field across the animal’s body. The PEMF device was calibrated to deliver a box waveform with 15 Hz frequency for 3 h per day, 7 days per week for 6 weeks. The study groups varied in magnetic field delivered: 0.41 mT, 1.2 mT, 4.1 mT, and 12.1 mT. The 1.2 mT group (30 T/s) demonstrated a reduction in trabecular bone loss by microCT at 6 weeks, similar to a control group treated with alendronate, but the 1.2 mT group did not reduce bone formation rates by dynamic histomorphometry assessment at 6 weeks as the alendronate control group did (*p* < .05). Of note, none of PEMF groups demonstrated a significant difference in bone mineral density by microCT at 6 weeks when compared to sham controls.

#### No Biomechanical Benefit Demonstrated in PEMF Small Animal Studies for Spinal Fusion and Implant Integration

In 1994, Guizzardi et al. demonstrated some benefit of PEMF in a rat posterior lumbar fusion model (Guizzardi et al., 1994). The PEMF device generated an induced electrical field within the cage of an average of 2.5 mV, but no other device specifications were offered. The animals in the stimulator group were exposed to PEMF for 12 h per day. In the sham stimulator group, typical regrowth of cartilage was observed by histomorphological analysis at 4 and 8 weeks. However, there was a noticeable, qualitative osteoblastic proliferation in the stimulator group, which was present at week 4 and increased at week 8. There was no biomechanical testing included in the protocol.

The use of PEMF to augment implant integration has also been explored. Buzza et al. used a custom PEMF device with a pulse width of 85 μs and 25 Hz frequency (the strength of magnetic field was not stated) on titanium dental implants in the tibiae of 12 rabbit (Buzzá et al., 2003). The legs of rabbits were placed within the coils for 30 min, 5 days per week, for 21 and 42 days, depending upon the group. When compared to the sham control group, there was no difference in torque required to remove the implants at either timepoint between groups.

#### A Variety of PEMF Protocols Demonstrats Some Benefit for Acute Fracture Management in Small Animals

Pulsed electromagnetic field therapy has also been tested in acute fracture models. In 2015, Atalay et al. demonstrated that PEMF induces osteogenesis in rat femur fractures (Atalay et al., 2015). Hammer osteotomies were performed with microplate fixation in 80 rats, and PEMF was administered around a small cage with a mean magnetic field of 1.5 ± 0.2 mT, 50 Hz for 6 h per day, 7 days per week for 30 days. In the PEMF group, there was significantly larger volumes of osteoblastic material by histomorphologic analysis on a 10-point scale than the sham control group at both 21 and 30 days post-operatively (*p* < .05). Liu et al. performed a similar study in which PEMF devices calibrated to sham, 1 mT, 5 mT, and 10 mT magnetic field strengths at 15 Hz were applied to three-month-old male Wistar rats in a 1 mm wide, unicortical femur bone wound model (Liu et al., 2021). On post-operative day 1, PEMF was applied for 2 h per day for 7 days. On experimental day 21, the bone mineral densities by microCT and fracture load were significantly higher in the 5 and 10 mT groups when compared to sham controls (*p* < .05). The 1 mT group was not statistically significant from the sham control group in any outcome measures tested. While Atalay et al. and Liu et al. both demonstrated benefit for acute bony injury healing with PEMF, the specifications and protocols vary significantly between studies.

#### PEMF Shown to Improve Ossification in Small Animal Distraction Osteogenesis Model

Fredericks et al. demonstrated a benefit of PEMF for distraction osteogenesis in a rabbit tibia model (Fredericks et al., 2003). After tibial osteotomies were performed, 0.25 mm distractions were carried out twice-daily for 21 days. The PEMF group was treated with 30 ms bursts at 1.5 Hz (no magnetic field strength was reported) for 1 h per day, 5 days per week for 21 days. At 16- and 23-days post-distraction, the PEMF group demonstrated statistically significant improvements in torsional strength, stiffness, and callus area ratios. A more recent study by Li et al., examined the effect of PEMF on distraction osteogenesis and angiogenesis in a rat tibial lengthening model (Li et al., 2020). Fifty-six rats were divided into two groups: distraction with sham stimulator and distraction with 30 T/s, 15 Hz PEMF treatment for 3 h per day for 4 weeks. Four-point biomechanical testing resulted in a larger force to failure (*p* < .05) and ultimate load (*p* < .01) when compared to sham controls at 4 weeks. Decalcified microCT at 1 and 3 weeks revealed a larger vascular volume/tissue volume ratio in the PEMF group (*p* < .05, *p* < .01, respectively). Li et al. supports the earlier study by Fredericks et al. in that the PEMF group developed more robust callus formation (Fredericks et al., 2003; Li et al., 2020), but Li et al. demonstrates the association between enhanced angiogenesis within the callus and PEMF therapy, which has not been previously reported.

### Capacitive Coupling Studies—Small Animal Studies

Although relatively few in number, four CC studies have been performed in small animals. In 1979, Marino et al. examined the effect of a 50 V/cm, 1590 V, 60 Hz CC device on rat fibular osteotomy healing (Marino et al., 1979). The rats in the treatment group were exposed to continuous, full-body, homogenous electrical field for 14 days. Compared to sham controls, the treatment group had significantly lower histomorphologic grades of healing at 21 days (*p* < .01). This study concluded that the low power, low frequency electrical field stunted the bone healing process in the treatment group.

#### Mixed Results Demonstrated for CC Small Animal Osteoporosis Models

The majority of CC studies in small animals to date have been to examine the effect on osteoporotic bone loss. In 1988, Brighton et al. demonstrated that CC may be used to treat disuse osteoporosis in a neurectomized rat hindlimb model (Brighton et al., 1988). Rats were labeled with tetracycline, sciatic-denervated, and treated with either a sham stimulator or a continuous 5 V, 60 Hz CC device over the tibia for 12 days either prior to developing osteoporosis or after osteoporosis was established. No electrical field strength was reported. By measuring incorporation of tetracycline, it was determined that the active stimulator group had less disuse osteoporosis when treated after the establishment of osteoporosis by means of increased bone formation (*p* < .01). However, CC did not affect the rate of bone resorption and was not effective in preventing osteoporosis. More recently, Medalha et al. used a spinal cord injury rat model to examine the effect of CC on disuse osteoporosis (Medalha et al., 2010). The spinal cord injured rats were treated with a CC device specified to deliver full-body 1.5 Mhz, 30 mW for 20 min, 3 times per week for 4 weeks. No voltage parameters were given, and no measurements of electrical field within the cage were described. Compared to controls, the CC group demonstrated no statistical difference in maximal load or densitometry but did show a smaller inner diameter and larger internal external area of the tibial diaphysis by morphometric analysis (*p* < .05). Another study demonstrated benefit of CC in an ovariectomized rat osteoporosis model (Manjhi et al., 2010). The active stimulator group received 10 V, 16 Hz stimulation fitted over the animals’ femur, 2 h per day for 60 days. No electrical field estimation was given. Bone mineral content and density were significantly higher in the active treatment group when compared to sham controls (*p* < .01, *p* < .001, respectively). No biomechanical protocols were performed.

## Large Animal Studies

Investigation into the efficacy of ES for the purpose of bone healing in a large animal model is a critical step in determining the optimal stimulation modality and specifications in humans. Large animal models offer more insight to a clinically useful bone growth stimulator—when compared to *in vitro* or small animal studies—as larger animals are more similar in scale and capacity for bone healing to humans (Table 3). Despite this, guidelines outlining optimal parameters for stimulator specifications and protocols have yet to be established. Trends within the large animal literature include failure of non-invasive modalities to induce osteogenesis as they did in small animal and *in vitro* models. Inconvertible and incomplete reporting of device specifications and lack of device function validation are also present as they were in small animals.

### Direct Current Studies–Large Animal Models

#### Early DCES Studies in Large Animals Demonstrating Improved Histologic, Radiographic Outcomes in Gap Nonunion

In 1977, Paterson et al. evaluated the use of DCES on tibial gap osteotomies of 69 dogs (Paterson et al., 1977a). Eight weeks post-osteotomy, a stainless-steel cathode was implanted within the 1.5 cm gap to provide a 20 μA constant current for 4 weeks. Histologic analysis completed at both 2 and 4 weeks demonstrated improved osteogenesis (*p* = .05), and radiographic assessment of union at 4 weeks showed improved healing (*p* = .05). Paterson published an additional study in 1977 evaluating the use of DCES in a tibial nonunion dog model (Paterson et al., 1977b). A battery-powered implant supplied a constant current of 20 μA to a 1.5 cm delayed union gap for 8 weeks following a 2-week healing period post-osteotomy. Histology and visual assessment of the callus was performed at both 2 and 4 weeks. At 4 weeks, the active treatment group demonstrated larger quantities of fibrous tissue and endochondral ossification by histology (*p* = .042), and visual assessment scores of bone healing were higher than controls (*p* < .01).

#### Acute Fractures, Spinal Fusion, and Autologous Bone Grafting Studied in Large Animal DCES Models With Varying Levels of Success

DCES has also been tested in large animal acute fracture models. The first study of this type used 20 μA constant DCES with stainless-steel cathodes embedded within long-leg plaster casts to treat tibial fractures of 28 dogs (Srivastava et al., 1982). Histomorphologic analysis at regular intervals revealed decreased time to fracture healing in the active stimulator group when compared sham controls, though no *p*-values were provided. In 1990, Chakkalakal et al. examined the relationship of different DCES amperage on dog radial fracture healing (Chakkalakal et al., 1990). Stimulation was delivered constantly via copper cathodes in groups of no stimulation (sham control), 1, 7, and 13 μA for 3 weeks. All active stimulator groups demonstrated improved rigidity by biomechanical testing (*p* < .001, *p* < .002, *p* < .009, respectively), as well as the mineral-to-matrix ratio by histomorphologic analysis (*p* < .021, *p* < .004, *p* < .025, respectively). There was no significant difference in outcomes reported between the 1,7, and 13 μA stimulator groups.

To evaluate the efficacy of DCES on spinal fusion, Nerubay et al. applied constant 20 μA stimulation with stainless-steel cathodes post-operative day 0–8 weeks in a porcine posterior lumbar fusion model on 30 animals (Nerubay et al., 1986). An increase in radiographic fusion score was noted at the 2-month mark compared to controls (*p* = .037). The active stimulation group also demonstrated an increase in osteoblastic activity by histomorphologic scoring at 1- and 2-month post-fusion (*p* < .01). Toth et al. evaluated the efficacy of continuous DCES for 4 months on sham control, 40 μA, and 100 μA groups (no cathode material reported) in a sheep posterior lumbar spinal fusion model (Toth et al., 2000). The mean radiographic fusion score increased in a dose-dependent manner with current, and the 100 μA group was significantly higher than sham controls (*p* = .003). Histologic grades of fusion also increased with current applied (*p* < .009, Fisher’s exact *t*-test). Biomechanical testing demonstrated an increased flexion-to-failure force in actively stimulated groups (*p* < .029). More recently, Cook et al. evaluated the effect of constant DCES on anterior spinal fusion in 35 *rhesus macaques* with titanium alloy interbody fusion devices (Cook et al., 2004). The experimental setup included groups of sham control, low current density (0.054 μA/mm^2^), and high current density (0.196 μA/mm^2^). Radiographs were completed at 4, 8, 12, and 26 weeks. The active stimulator groups demonstrated a reduced time to fusion by radiographic fusion grade (*p* = .0001). At 26 weeks, computed tomography demonstrated a higher fusion rate in the active stimulator groups (*p* = .0314).

Lindsey et al. evaluated the effect of DCES on biomechanical outcomes for autologous bone grafts in a dog femur model (Lindsey et al., 1987). Using a coiled titanium cathode, 20 μA was delivered to the graft site constantly for 8 weeks. Of note, no description of the electrode size or surface area was offered. No difference in torsional force-to-failure between groups was observed (*p* > .669). The results of this study differ from the spinal fusion studies (Nerubay et al., 1986; Toth et al., 2000; Cook et al., 2004) despite the clinical similarities of using DCES to augment healing of autologous bone graft within a defect.

### Pulsed Electromagnetic Field Studies—Large Animal Models

#### No Benefit Shown for Large Animal Bone Grafting Treated With PEMF

In addition to DCES, PEMF has been tested for a variety of indications in a large animal model. In 1984, Miller et al. evaluated the effect of PEMF in a segmental autologous cortical bone grafts in the fibulas of 20 dogs (Miller et al., 1984). Custom designed orthoses incorporated the PEMF device, specified for 15Hz, 5 ms bursts in an asymmetrical waveform for 20 h per day. No magnetic field strength was reported. There was no significant difference between sham and active stimulators for biomechanical testing or histomorphologic assessment of osteoproliferation.

#### PEMF Largely Ineffective at Improving Outcomes in Large Animal Model Gap Osteotomies

In 1985, Law et al. investigated the effects of continuous 1.1 mT PEMF treatment for 6 weeks on the rate of healing of transverse, gapless tibial osteotomies in 26 sheep (Law et al., 1985). At 2- and 6-week post-osteotomy, histology staining measured cortical bone and bone callus formation, while radiographs measured the total mineralized callus. No significant improvement in bone healing was found in any of the variables investigated. Inoue et al. investigated the effects of PEMF on late bone healing phases using an osteotomy gap model (Inoue et al., 2002). Twelve dogs underwent a transverse mid-diaphyseal tibial osteotomy with a 2 mm gap. PEMF (calibrated for 0.2 mT, 1.5 Hz) was applied for 1 h a day beginning 4 weeks after the osteotomy and continued for an additional 4 weeks. Biomechanical testing at 12 weeks revealed significantly increased maximum torque in the PEMF group (*p* < .04). Histomorphologic scoring at 8 and 10 weeks post-operatively was significantly increased compared to the control group (*p* < .04, *p* < .05, respectively). Inoue et al. have posited that the timing of ES initiation may play a role in significant improvement as their model showed an improvement in bone formation with ES beginning 4 weeks status post osteotomy, while Law et al. initiated ES immediately post-operative day zero (Law et al., 1985; Inoue et al., 2002).

#### Capacitive Coupling Studies—Large Animal Models

Relatively few CC studies have been performed in a large animal model. Pepper et al. examined the effect of CC on a canine tibial distraction osteogenesis model (Pepper et al., 1996). The animals underwent tibial osteotomy, followed by a 5-day period of healing. At this time, they underwent distraction at 1 mm/day for 21 days. At the completion of distraction, they were randomized to sham or active CC with 60 kHz, 3–6.3 V, 5–10 mA for 28 days. No electric field strengths were reported. Biomechanical, histologic, and radiographic analyses revealed no statistical difference between groups. Muttini et al. investigated the efficacy of CC in a sheep tibial delayed union model (Muttini et al., 2014). The device was calibrated to 1,500 μA, 12.5 Hz with a duty cycle of 50% for 40 ms for 12 h a day. No electric field strengths were reported. Callus maturation was found to be more robust in the active treatment group between 30–45 days postoperatively by quantitative histomorphometry (*p* < .0001), and quantitative radiodensity analysis demonstrated increased opacity in the active stimulation group (*p* < .0043). Of note, the CC device in this study was attached to the stainless-steel pins of an external fixator immediately proximal and distal to the osteotomy, which acted as the electrodes for the alternating current.

## Discussion

### Roadblocks to Clinical Translation

**FIGURE 1:**
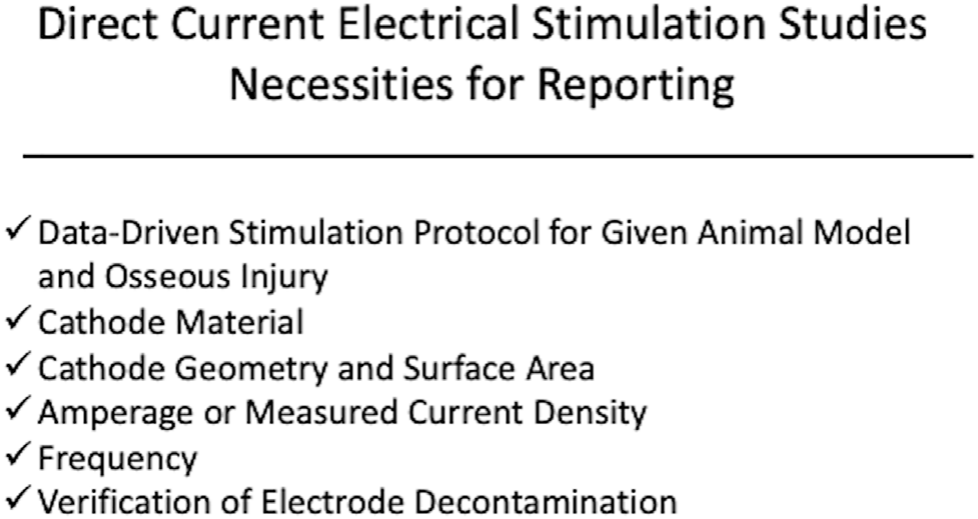
Displayed is a schematic demonstrating the recommended components for device specifications and stimulation protocol for a preclinical direct current electrical stimulation (DCES) study. The authors recommend reporting cathode material, current density, cathode geometry, frequency, and verified electrode decontamination and applying a stimulation protocol based on successful outcomes from prior studies within the context of the osseous injury being studied.

**FIGURE 2:**
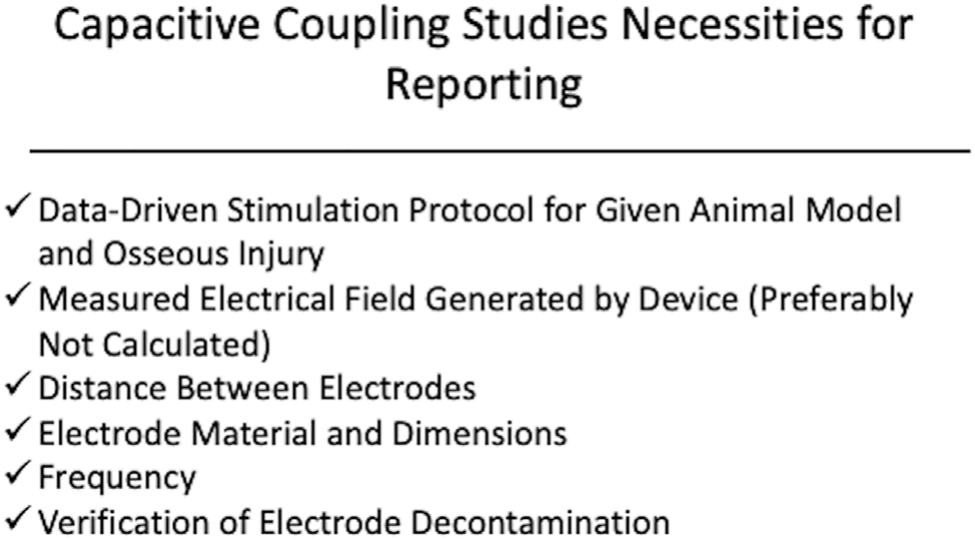
Displayed is a schematic of the recommended components of device specifications and stimulation protocol for capacitive coupling. The authors recommend reporting a measured electric field strength, frequency, distance between electrodes, electrode material, and verification of electrode decontamination. It is also recommended that the stimulation protocol be derived from previous studies demonstrating benefit of the ES modality within the context of the osseous injury being studied.

Despite success in preclinical models for EBGSs, a recent meta-analysis of sham-controlled, randomized clinical trials have shown that EBGSs in humans demonstrate mixed results (Aleem et al., 2016). While the lack of clinical translation in this field is likely multifactorial, significant issues with preclinical studies that impede translation include the lack of repeatability and consistency. In many studies, the device specifications are incomplete, which makes it impossible to repeat the study or apply the stimulation parameters to another model (Miller et al., 1984; Brighton et al., 1988; Guizzardi et al., 1994; Shankar et al., 1998; Toth et al., 2000; Buzzá et al., 2003; Fredericks et al., 2003; Muttini et al., 2014; Dauben et al., 2016; Jing et al., 2019). This is especially important because many of the studies use custom stimulators. For a DCES model, we would recommend reporting the cathode material, current density (or raw current with cathode surface area), and frequency (Figure 1). Spadaro demonstrated that different cathode materials induce osteogenesis at different current densities (Spadaro, 1982). Direct current is believed to induce osteogenesis via an electrochemical reaction where the cathode catalyzes the reduction of cellular oxygen to free radicals, generating an alkaline and relatively low oxygen microenvironment, favoring osteoclast-derived vascular endothelial growth factor synthesis and increasing osteoblast to osteoclast ratio (Brighton and Friedenberg, 1974; Brighton et al., 1975; Bushinsky, 1996; Cho et al., 2001). For this reason, the same raw current applied to two different cathode materials may not yield the same results. The same is true with current density. This is illustrated by comparing the results of Spadaro et al. and Shafer et al. (Spadaro, 1982; Shafer et al., 1995). Spadaro et al. successfully induced osteogenesis with 5 μA applied to a titanium wire cathode, yielding a current density of 0.2 μA/mm^2^ (Spadaro, 1982). Shafer et al. applied 7.5 μA to a titanium dental implant with a surface area of 104.55 mm^2^, yielding a calculated current density of 0.07 μA/mm^2^ (Shafer et al., 1995). Shafer et al. concludes that DCES is not effective in improving dental implant osseointegration, but Spadaro et al. previously demonstrated that titanium cathodes require higher current density than what was applied in Shafer et al. to be effective. Therefore, it is critical to include current density (or sufficient information for calculating it) and cathode material in the methods of a DCES study. In the same vein, the authors would recommend performing bench tests on all electrodes used in DCES or CC studies prior to use to ensure they are free of surface contaminants, negatively affecting the electrochemical reactions occurring at the surface of the electrode (DCES) and the resultant electrical field (CC) (Yamano et al., 2011). For CC studies, the minimum reporting parameters for a reproducible study are measured electrical field, electrode dimensions and material, frequency, and distance between electrodes (Figure 2). The main limitations for CC are a function of distance from the electrode and intervening insulative soft tissue, as the fall off of an electric field from the signal source is very steep, ∼1/*r* ^2^ where r is the distance from the electrodes (Plonsey and Barr, 1995). Therefore, when scaling up to larger animal or human, a much larger electrical field is required to reach the osseous injury.

**FIGURE 3:**
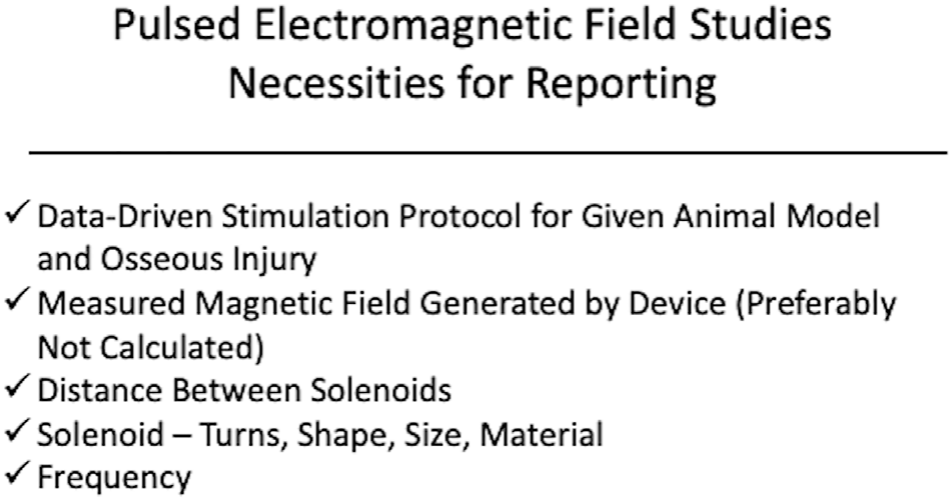
Displayed is a schematic of the recommended components of device specifications and stimulation protocol for pulsed electromagnetic frequency. The authors recommend reporting a measured magnetic field strength, frequency, and solenoid parameters (turns, size, shape). It is also recommended that the stimulation protocol be derived from previous studies demonstrating benefit of the ES modality within the context of the osseous injury being studied.

Some PEMF studies report combinations of pulse parameters, frequency, amperage, and voltage of a custom PEMF device, but this information is not sufficient to recreate the PEMF device. Very few studies report the device geometry. For PEMF devices, it is recommended to report solenoid turns, shape, size, and distance between coils. These variables all affect the induced electric field and are necessary to recreate the device (Lunt, 1985). When translating results of PEMF studies to large animals and humans, the increased scale and thickness of tissue layers require alterations to coil distance, turn number, amperage, and voltage to maintain a conserved magnetic field strength at the level of the osseous injury (Lunt, 1985). Therefore, in addition to reporting the specifications aforementioned, it is recommended to experimentally measure the induced electric or magnetic field generated by the custom device as opposed to calculating it (Figure 3). Without these critical pieces of information, little can be gained from the experimental outcomes.

The stimulation protocols from study to study also vary significantly. This is in part due to the various indications and animal models to which ES has been applied. For example, it is logical to apply DCES in a large animal spinal fusion model for much longer than a small animal acute fracture model, based on the amount of time expected to heal the respective osseous injuries. However, it remains to be elucidated at what point in the bone healing process—hematoma formation, callus formation, and osseous remodeling—that ES is most beneficial. Inoue et al. offers some information that perhaps initiating PEMF therapy at the late remodeling phase of bone healing is superior starting post-injury day zero (Inoue et al., 2002). Additionally, there is no consensus on the amount of time per day ES should be applied. This is not a problem for DCES therapies, as many protocols stimulate constantly for the duration of the study. However, for removable noninvasive EGBSs, this is both an inconsistency in preclinical studies and a problem for patient compliance. Studies that have tried to determine the optimal time per day for PEMF therapy have been unsuccessful. For example, Van der Jagt et al. stimulated groups of rats in an osteoporosis model with 2 and 4 h per day of PEMF therapy, but no treatment group demonstrated a benefit (van der Jagt et al., 2012). To confuse the picture further, Miller *et al.* was not successful in inducing osteogenesis with 20 h per day of PEMF in a dog autologous bone graft model, whereas Inoue et al*.* demonstrated a biomechanical benefit of PEMF for 1 h per day in a dog osteotomy model (Miller et al., 1984; Inoue et al., 2002). These study results highlight that there are currently no uniform guidelines for time per day of PEMF therapy.

Inconsistency in reporting and outcome measures also make the results of studies difficult to compare. *In vitro* studies have been performed on various cell lines—osteoblasts, chondrocytes, osteoclasts, mesenchymal stem cells—of both animal and human origin. In each of these studies, different cellular markers of osteogenesis are used. The most commonly described markers are ALP, COL-1, and measurements of metabolic activity, so we would recommend including these in *in vitro* studies to optimize direct comparison. For animal studies, heterogeneity and lack of reporting on the specifications of experimental animals and osseous injuries make it difficult to compare results of studies. For this reason, detailed descriptions in orientation and gap size if applicable are recommended. Additionally, biomechanical testing is the gold standard outcome. However, many studies report purely histomorphologic or radiographic outcomes. These methods of measuring bone healing are useful secondary markers of bone strength, but we would recommend using them as adjuncts to biomechanical testing, as this is the empirical, clinically relevant measurement of bone healing.

### The Issue of Getting Stimulation to the Osseous Injury

**FIGURE 4:**
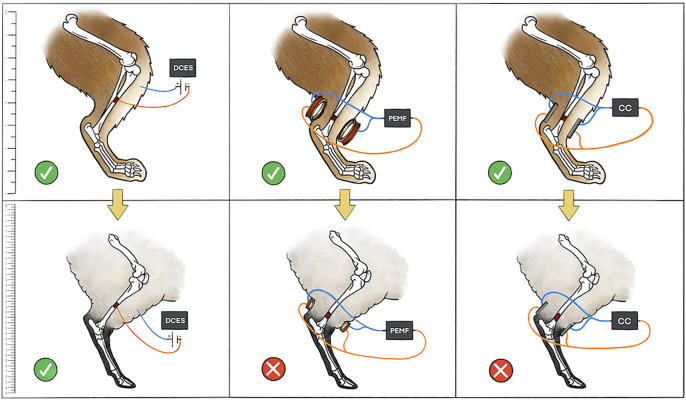
Depicted is a pictorial schematic of the three types of ES discussed in the manuscript in both a small and large animal model. The small animal model (rabbit) is in the top panels with a yellow arrow symbolizing translation to a large animal model (sheep) in the bottom panels. A green check means that the ES was successful in stimulating bone growth, whereas a red “X” means that the ES treatment was not successful. The authors attribute issues of scale with CC and variations in limb soft tissue and bone thickness with PEMF to the lack of success of non-invasive ES modalities in large animals. Scale bar on left for reference.

When examining this body of literature, there are general trends that become apparent. Direct current electrical stimulation, CC, and PEMF therapies have all demonstrated benefit in cell signaling within the osteogenesis pathway in multiple cell lines. This has translated well into the small animal model where there has been histomorphologic evidence of improved osteogenesis with all ES therapies. There has also been robust radiographic and biomechanical evidence supporting all types of ES in small animals. However, the large animal studies demonstrate a stark contrast between the invasive and noninvasive EGBSs. While the majority of DCES studies in large animals yielded positive results, the non-invasive modalities—PEMF and CC—were generally less successful in all outcome measures studied. Reasons to explain this finding include issues with delivery of stimulation to the fracture site. The amount of soft tissue, thickness of the bony cortex, and width of bone all significantly affect the induced electrical field generated by PEMF (Lunt, 1985). These modeling data can explain why greater success is seen with DCES studies, where current is applied directly to fracture sites in large animals (Figure 4). Similarly, there is a steep fall-off of CC electrical field that makes it difficult to stimulate osseous injuries within larger limbs (Plonsey and Barr, 1995). This is reflected in the literature as a single large animal CC study demonstrated benefit (Muttini et al., 2014)*.* The authors showed an improvement in histomorphologic and radiographic evidence of osteogenesis in a sheep tibial delayed union model. It is interesting that in this experimental setup the stainless-steel pins of the external fixator acted as the electrodes for the CC device. It is possible that Muttini et al. were successful in inducing osteogenesis because the of the pins bypassed intervening soft tissue that would be present in a standard, non-invasive CC device design (Muttini et al., 2014).

The lack of success seen with non-invasive modalities in large animals is also seen in human studies. A recent literature review of all randomized, sham-controlled trials of ES for the purpose of healing acute fractures in humans was conducted (Nicksic et al., 2021). Of the five studies that met inclusion criteria, four used PEMF, and one used DCES. The only study that demonstrated a significant reduction in time to radiographic union was the invasive, DCES study (Itoh et al., 2008). In conjunction with the large animal data, these results highlight that there are issues with translation of non-invasive modalities of ES to larger limbs. Stricter adherence to reporting critical device specifications and stimulation protocols in preclinical studies, as well as increasing scientific rigor—blinding, sham controls, appropriate power, verifying baseline device function experimentally—is the first step to translating the success seen in small animal and *in vitro* studies. Another important recommendation is that researchers pre-register all *in vitro* and animal studies. Open Science Framework and the Animal Study Registry are two options for pre-registration of preclinical studies (Bert et al., 2019). Disclosing the research strategy ahead of the experiment defines interventions and outcome measures while creating a Digital Object Identifier number to protect intellectual property. Pre-registration will further decrease reporting bias and distinguish hypothesis testing from exploratory research, a problem that becomes especially significant when confirmatory statistical inference is applied to exploratory discoveries (Nosek et al., 2018).

## Conclusion

Despite the wide range outcome measures, device specifications, and stimulation protocols, conclusions can be drawn from examining the preclinical body of literature for EBGSs. *In vitro* studies have demonstrated significant effects on a multitude of cellular markers of osteogenesis in multiple different cell lines, using DCES, CC, and PEMF. These cellular markers of proliferation are corroborated in small animal studies, many of which were successful in demonstrating histomorphologic evidence of improved bone healing. However, in large animal studies, the invasive modality of ES, DCES, demonstrated more impressive results in radiographic, histomorphologic, and biomechanical outcomes. This may be in part due to issues with delivery of ES to the site of bony injury in larger animal models. Further modeling data is required to demonstrate that CC and PEMF devices are delivering consistent electrical fields at the level of the fracture site in large animals. This will help guide clinically applicable specifications for these devices.
